# Validity, reliability, and responsiveness of the Swedish version of Western Ontario Osteoarthritis of the Shoulder index

**DOI:** 10.1186/s12891-022-05300-1

**Published:** 2022-04-11

**Authors:** Kristofer Hallberg, Björn Salomonsson

**Affiliations:** 1 Department of Clinical Sciences, Karolinska Institutet, Danderyd Hospital, Danderyd, Sweden; 2 Division of Orthopaedic Surgery, Danderyd hospital, Stockholm, Sweden

**Keywords:** Patient reported outcome measures, Validation study, Arthroplasty, Replacement, Shoulder

## Abstract

**Background:**

The Western Ontario of the Shoulder index (WOOS) is a patient-reported, disease-specific instrument, designed to measure quality of life in patients with osteoarthritis of the shoulder. The Swedish Shoulder Arthroplasty Registry (SSAR) uses WOOS and EuroQoL 5-dimensions 3 levels (EQ-5D-3L) as patient reported outcome measures. The purpose of this study was to test the validity, responsiveness, and reliability of the Swedish translation of WOOS for patients with osteoarthritis of the shoulder.

**Methods:**

Data was collected from three shoulder arthroplasty studies performed during 2005–2013, with 23, 21, and 19 patients respectively. Forms were collected preoperatively, and postoperatively between 12 and 24 months. WOOS and EQ-5D-3L were used in all three studies. Additionally, the Oxford Shoulder Score (OSS) (*n* = 23) was used in one study, and the Constant-Murley score (CMS) (*n* = 40) in two of the studies. Validity was analysed by calculating Pearson’s correlation coefficient (PCC). Cronbach’s alpha (CA) was used to estimate internal consistency and reliability. The responsiveness of WOOS was measured by calculating effect size and standardized response mean. To assess the performance of WOOS over time, we present repeated measures of WOOS in the registry over a 10-year period.

**Results:**

The validity analysis showed excellent correlations of WOOS to CMS, OSS and EQ-5D 3L, with Pearson’s correlation coefficient of 0.72, 0.83, and 0.62 respectively (*P* < 0.001). There were adequate floor effects in the sport and lifestyle domains preoperatively, and adequate ceiling effects in all domains postoperatively. There were no floor effects and adequate ceiling effects for total WOOS. Analyzing reliability, Cronbach’s alpha was 0.95 for the pre- and postoperative WOOS scores combined. The analysis of responsiveness for WOOS showed an effect size of 2.52 and a standardized response mean of 1.43.

The individual results measured by WOOS within the registry shows stable levels from 1 to 10 years.

**Conclusion:**

The Swedish translation of WOOS is valid, reliable, and responsive for use in a clinical setting for patients with glenohumeral osteoarthritis treated with shoulder arthroplasty, and we regard it as an appropriate instrument for use in the Swedish Shoulder Arthroplasty Registry.

## Background

Patient-reported outcome measures (PROM) are frequently used for evaluating treatment in orthopedic surgery, and many disease-specific measures have been developed to reach a higher level of responsiveness and specificity than a more general Quality-of Life (QoL)-score might do.

The most frequently used shoulder evaluation tools were developed in English [1–3] and are used in many countries with different languages and cultural traditions. It is important that these tools are translated and adapted to the context in which they are to be used and done according to internationally accepted and standardized guidelines [4, 5]. The Swedish language is spoken by more than 13 million people, mainly in Sweden but to some extent also in Finland. It is the first language for a population of approximately 10 million. The Swedish Shoulder Arthroplasty Registry (SSAR) was started in 1999 and contains data on more than 20,000 shoulder arthroplasties. It has a coverage of more than 90% of the shoulder arthroplasties performed in Sweden.

SSAR uses the Western Ontario Osteoarthritis of the Shoulder index (WOOS) and EuroQol 5-dimensions (EQ-5D) as patient reported outcome measures. For orthopedic registries, it is desirable to monitor pain, function, and general health status before and after joint replacement surgery. A broad monitoring may improve our understanding about timing of surgery, arthroplasty indication, trajectories of patients who are not candidates for joint replacement, and factors associated with successful disease management [6]. Another important property for evaluation tools for registry use is that the patients can report their outcome in a practical manner, preferably without a clinical examination. The Swedish shoulder arthroplasty registry currently use paper-forms of WOOS, EQ-5D and satisfaction level, distributed by mail.

The Nordic registries have all agreed on WOOS as a PROM to use for evaluation of clincal results after shoulder arthroplasty, as this will make comparisons between countries easier and also facilitate pooling of results from the different countries. Of the shoulder specific instruments in this study, WOOS is the only one containing an emotional domain.

The purpose of our study was to test the validity, responsiveness and reliability of the Swedish translation of WOOS used within the SSAR, as well as to correlate the WOOS to EQ-5D 3L and OSS, which are PROM frequently in use by other national registries. We also wanted to correlate WOOS to CMS, a score often used in clinical studies, to facilitate future comparisons of outcomes, and to report the minimal detectable change (MDC) and minimum clinically important difference (MID) for WOOS.

## Methods

### Western Ontario Osteoarthritis of the Shoulder index

WOOS was developed by Lo et al. in 2001 at the University of Western Ontario, as a disease-specific measurement tool for shoulder related Quality-of-Life (QoL) [1]. It was designed for use as a PROM when evaluating different treatment regimens for patients with glenohumeral osteoarthritis (OA). The WOOS index has previously been translated into Swedish and its validity tested on patients with subacromial pain [7]. The WOOS score consists of 19 items divided into four domains: there are 6 items in physical domain, 5 items in the sport domain, 5 items in the lifestyle domain and 3 items in the emotional domain. The items are answered on a 0–100 mm visual analog scale (VAS), ranging from least to worst symptoms. The total score ranges from 0 (best) to 1900 (worst). The total score may be recalculated as a percentage, where a WOOS% of 100% represents a completely healthy shoulder. Since 2004, WOOS is used in the Swedish shoulder arthroplasty registry for pre-operative assessment, and follow-up at 1, 5 and 10 years, and presented as WOOS%. It is also used in the other Nordic shoulder registries.

### Constant-Murley Score

Constant-Murley Score (CMS) was published by Constant and Murley in 1987 [3]. It is a health instrument specifically developed for functional assessment of the shoulder and combines a physical examination with a questionnaire. A maximum of 100 points reflects a fully functional painless shoulder. The score is composed of four domains, all of which have different maximum points. The domains represent pain (15 points), activities of daily living (20 points), range of motion (40 points) and strength (25 points). The range of motion, and strength, of the shoulder should be assessed by an independent examinator, and the CMS is therefore not a true PROM, but adaptations has been proposed for CMS to be used as a patient self-reporting instrument [8].

### EuroQol 5-dimensions 3 levels

EuroQol 5 dimensions 3 Levels (EQ-5D-3L) is a generic health instrument that measures quality-of-life, (QoL). Health is assessed in five dimensions: mobility, self-care, usual activities, pain or discomfort, and anxiety or depression [9]. Each of the dimensions is divided in three levels: no problems, some or moderate problems and extreme problems. EQ-5D also includes a self-rating of health status on a 20 cm vertical VAS scale.

### Oxford Shoulder Score

Oxford Shoulder Score (OSS) was originally published in 1996 as a 12-item questionnaire [10]. It was developed for patients treated with shoulder surgery (other than stabilizing surgery). Each item is scored on a Likert scale, giving points of value from 1 to 5, i.e., from least to most difficulty or severity. The results of the individual items are then added to produce a total score ranging from 12 (least difficulties) to 60 (most difficulties, 60 worst result). OSS has later been adjusted to be calculated and presented as a score between 0–48 (48 best result) [11]. OSS is used as PROM for shoulder arthroplasty within the New Zealand and the United Kingdom National Joint Registry.

The material used in this study was collected from three previous studies at the Orthopaedic department at Danderyd hospital during the years 2005–2013. It consists of preoperative and postoperative (12 – 24 months) PROM from patients who underwent shoulder arthroplasty. The material was collected in three separate groups as described below. Statistical analyses were performed both using the separate groups and the three groups pooled (Table 1).

**Table 1:** Scores completed by patients in groups A, B and C

	**Group A**		**Group B**		**Group C**	
	**Preop**	**Postop**	**Preop**	**Postop**	**Preop**	**Postop**
**WOOS(n)**	23	23	21	21	19	17
**CMS(n)**	-	-	22	19	17	15
**OSS(n)**	14	14	-	-	-	-
**EQ-5D 3L(n)**	23	23	21	18	19	17

*WOOS* Western Ontario Osteoarthritis of the Shoulder score, *OSS* Oxford Shoulder Score, *CMS* Constant Murley Score, *Preop* patients who completed preoperative scoring, *Postop* patients who completed postoperative scoring

#### Group A (*n* = 23)

Group A consisted of 4 men and 19 women (44–85 years) treated with anatomical shoulder arthroplasty during the period January 2005 to March 2006 at Danderyd hospital**.** All of the patients completed the WOOS and EQ-5D preoperatively and at 12 months postoperatively. Fourteen patients also completed the OSS both preoperatively and at 1 year postoperatively.

#### Group B (*n* = 21)

Group B consisted of 13 men and 10 women (51- 81 years), treated with humeral head resurfacing hemi arthroplasty during the period January 2009 to August 2010 at Danderyd hospital. Scoring was done preoperatively, and at 12 and 24 months postoperatively. The scoring systems used were WOOS, CMS, and EQ-5D. 22 patients completed the CMS, 20 completed WOOS and 21 completed EQ-5D preoperatively. 19 patients completed the CMS, 21 completed WOOS and 18 completed EQ-5D postoperatively. 3 patients did not undergo the planned surgery but completed all three scores preoperatively.

#### Group C (*n* = 19)

Group C consisted of 5 men and 14 women, age between 50 and 74, treated with humeral head resurfacing during the period January 2012 to June 2013 at Danderyd hospital. Scoring was done preoperatively and at 3 months postoperatively. Scoring systems used were WOOS, CMS and EQ-5D 3L. 17 patients completed the CMS, and all 19 patients completed the WOOS and EQ-5D preoperatively. 15 patients completed the CMS, and 17 patients completed the WOOS and EQ-5D postoperatively. Two patients did not undergo the planned surgery but completed all three scores preoperatively. Two patients had missing data on the CMS both pre- and postoperatively.

#### WOOS performance over time in the registry

Within the SSAR almost 20,000 primary shoulder arthroplasties was reported 1999–2020. Approximately 7500 were diagnosed as primary OA, and 1300 as secondary OA. In 2004 the SSAR started to register a voluntary pre-operative score. In this article we present an additional 119 shoulders from the registry with primary (*n* = 103) and secondary (*n* = 16) OA diagnose which at the time of the study had reported all four PROM assessments (pre-op, 1, 5 and 10 years). This group consisted of 72 total shoulder arthroplasties, 26 stemmed hemi arthroplasties, and 21 resurfacing arthroplasties. The analysis was made to display the performance over time of the PROM.

### Statistical methods (Table 2)

**Table 2:** Statistical methods and their interpretations [12, 13]

	**Excellent**	**Adequate**	**Poor**
Cronbach´s α	≥ 0.80	0.70–0.79	< 0.70
PCC	≥ 0.60	0.30–0.59	< 0.30
Floor and Ceiling effects	No effects	≤ 20%	> 20%
ES and SRM	≥ 0.80	0.50–0.79	< 0.50

*PCC* Pearson’s correlation coefficient, *ES* effect size, *SRM* standardized response mean

Descriptive data is presented as per cent or absolute numbers, and as mean value with standard deviations when appropriate. The sample size exceeded a subject to item ratio of 3 for WOOS, which we deemed as sufficient for the validation analyses [14].

#### Validity

Convergent criterion validity was analyzed by calculating Pearson’s correlation coefficient (PCC). PCC can vary between -1 and 1, where 1 represents a complete correlation, 0 represents no correlation, and -1 a complete inverted correlation. The content validity was analyzed calculating the floor and ceiling effects for WOOS. In this study, a WOOS% score of 0–2% was considered a floor value, and a score of 98–100% was considered a ceiling value of the 0–100% in an item of the WOOS%. Floor and ceiling effects was calculated for groups A, B and C combined.

#### Reliability

Internal consistency reliability was used as an estimate of how well the items in a PROM yield consistent scores. It is desired for scores on similar items to be related, and at the same time contribute with some unique information. Cronbach’s alpha (CA) was used to estimate the reliability of internal consistency. CA can take on values between 0 and 1, and a value greater than 0.7 was considered adequate [15]. CA will increase when correlations between items increase. A value of 1 should be interpreted as a complete correlation between items. This is undesirable because it means that the items are too similar, and thus redundant [16].

#### Responsiveness

The responsiveness was calculated by using effect size (ES) and standardized response mean (SRM). ES was calculated by dividing the difference between a pre- and postoperative score by the preoperative standard deviation. SRM was calculated similarly, but the difference was divided by the postoperative standard deviation instead. A high value in both cases represents high responsiveness of the instrument. Values above 0.8 were considered excellent. ES and SRM were calculated for groups A, B and C combined.

#### Minimal detectable change (MDC) and minimum clinically important difference (MCID)

The minimum amount of change in an individual score that is not a measurement error, the MDC, was calculated using the Standard error of measurement (SEM). MDC = 1.96 × SEM x square root of 2. To specify a MCID for interpreting mean differences, the MCID was defined using a distribution-based approach, as 0.5 times the standard deviation [17].

All statistical analyses were performed in SPSS. Descriptive statistics were used to present minimum, maximum, and mean values for WOOS, as well as pre- and postoperative standard deviation. A *p*-value less than 0.05 was chosen to determine significance.

## Results

### Convergent criterion validity

The correlations between WOOS and CMS, OSS and EQ-5D 3L respectively, were all significant (*P* < 0.001) (Table 3). The correlation between OSS and EQ-5D 3L was also high (*P* < 0.001). The correlation between CMS and EQ-5D 3L was lower (PCC = 0.37, *P* = 0.003). No correlation analysis between CMS and OSS was performed, because no patient group had been assessed with both these questionnaires.

**Table 3:** Correlation coefficients between the Western Ontario Osteoarthritis of the Shoulder (WOOS) index and the CMS, OSS and EQ-5D 3L scoring systems. All the correlations were significant (*P* < 0.05)

	**WOOS**	**CMS**	**OSS**	**EQ-5D 3L**
WOOS	-	0.72	0.83	0.62
CMS	0.72	-	-	0.37
OSS	0.83	-	-	0.70
EQ-5D 3L	0.62	0.37	0.70	-

*WOOS* Western Ontario Osteoarthritis of the Shoulder score, *OSS* Oxford Shoulder Score, *CMS* Constant Murley Score

To investigate further the correlation between WOOS and EQ-5D 3L, correlation analyses between the different WOOS domains and EQ-5D were performed.

Correlations were also made between WOOS and the clinically examined items of CMS, to investigate if WOOS was able to capture results from these items of CMS (Table 4).

**Table 4:** Correlation coefficients (PCC) between WOOS domains and EQ-5D 3L or clinically examined items of CMS. All correlations were significant (*P* < 0.05)

WOOS domain	EQ-5D 3L	CMS
Total	0.62	0.53
Physical	0.60	0.59
Sport	0.55	0.44
Lifestyle	0.57	0.56
Emotions	0.51	0.20

### Content validity

There were adequate floor effects in the sport and lifestyle domains preoperatively, and adequate ceiling effects in all domains postoperatively. There were no floor effects and adequate ceiling effects for the total WOOS (Table 5).

**Table 5:** Floor and ceiling effects both pre- and postoperatively for the WOOS domains and the total WOOS. Percent of patients within limits for floor and ceiling effects. (See Methods section for definition of floor- and ceiling effect)

Domain	Floor		Ceiling	
	**Preop**	**Postop**	**Preop**	**Postop**
**Physical**	-	-	-	3.3%
**Sport**	3.0%	-	-	4.9%
**Lifestyle**	1.6%	-	-	3.3%
**Emotions**	-	-	-	13.1%
**WOOS total**	-	-	-	3.3%

### Reliability

The reliability of the internal consistency was tested by calculating Cronbach’s alpha for separate domains as well as for all domains combined to a total WOOS (Table 6). For pre- and postoperative scores combined, CA exceeded 0.8 in all domains. The total score showed a higher CA than any of the separate domains. The same results were shown when calculating CA on postoperative scores alone. In the preoperative scores, CA was lower in all domains as well as for the total score. The CA in the sport, lifestyle and emotions domains did not exceed 0.8. The sport and lifestyle domains showed CA below 0.7, which is regarded as poor. The emotions domain had a CA of 0.72.

**Table 6:** Cronbach’s alpha for the different WOOS domains, pre- and postoperatively as well as combined. All calculations were significant (*P* < 0.05)

Domain	Preop	Postop	Preop + postop
Total	0.81	0.95	0.95
Physical	0.81	0.84	0.85
Sport	0.63	0.89	0.87
Lifestyle	0.69	0.89	0.90
Emotions	0.72	0.88	0.85

### Responsiveness

We calculated both ES and SRM to test responsiveness. SRM was calculated to ease comparison with studies where SRM was calculated instead of ES. All scoring systems exceeded 0.8 in ES. The WOOS score showed the highest ES with 2.52, while EQ-5D showed the lowest ES with 0.82 (Table 7).

**Table 7:** Minimum, maximum and mean values as well as effect size and standardized response mean for the different scoring systems

	**Min**	**Max**	**Mean**	**ES**	**SRM**
WOOS preoperative	657	1709	1153	2.52	1.43
WOOS postoperative	5	1533	550		
CMS preoperative	19	55	33	1.50	1.10
CMS postoperative	19	70	49		
OSS preoperative	29	53	41	1.68	1.45
OSS postoperative	16	42	28		
EQ-5D preoperative	- 0.02	1.00	0.41	0.82	0.86
EQ-5D postoperative	- 0.35	1.00	0.67		

*ES* effect size, *SRM* Standardized Response Mean, *Min* Lowest score, *Max* Highest score, *WOOS* Western Ontario Osteoarthritis of the Shoulder scorem, *OSS* Oxford Shoulder Score, *CMS* Constant Murley Score

**Fig. 1:**
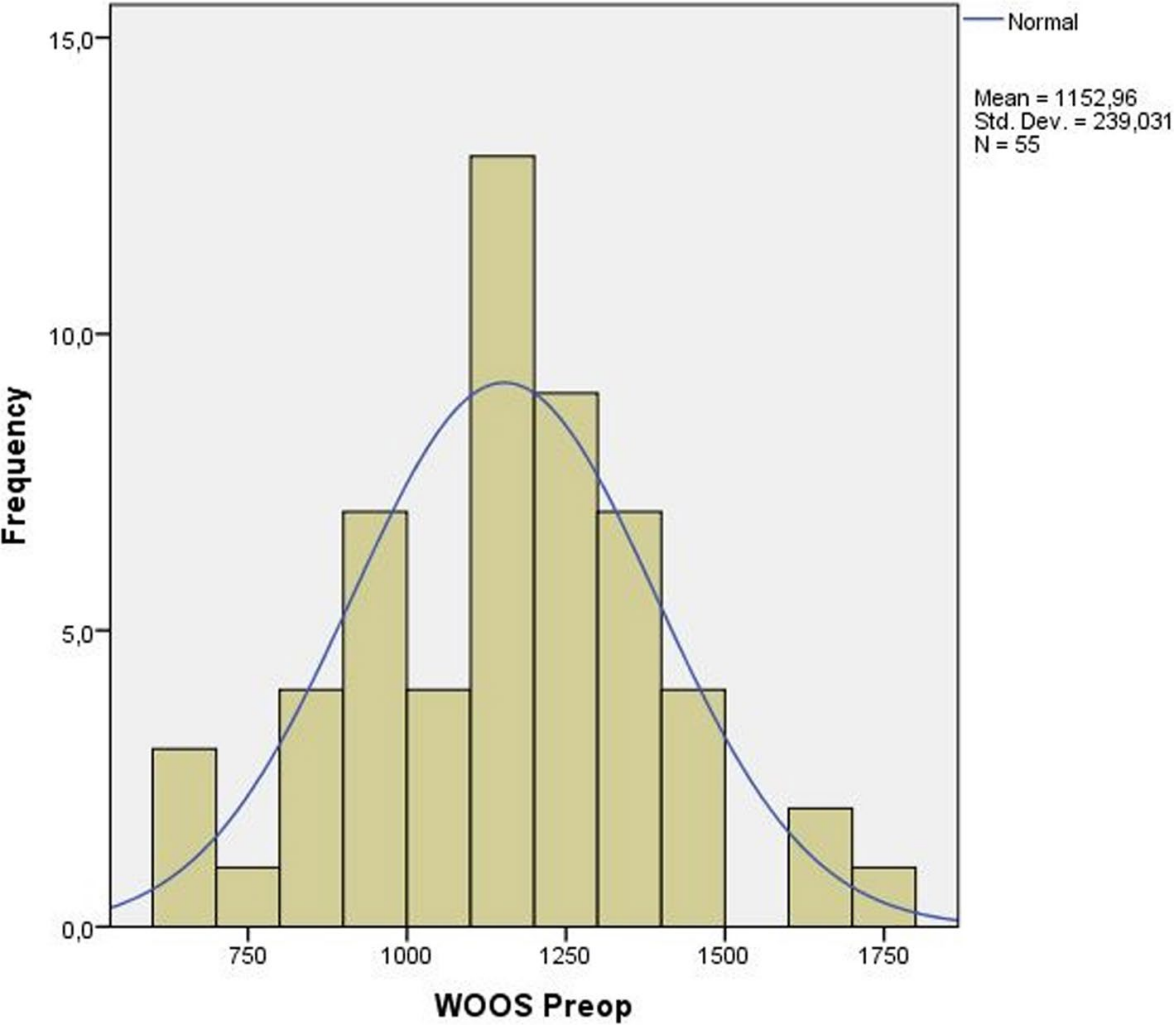
Preoperative WOOS scores presented as a histogram. X-axis=WOOS-score, Y-axis= Number of subjects

**Fig. 2:**
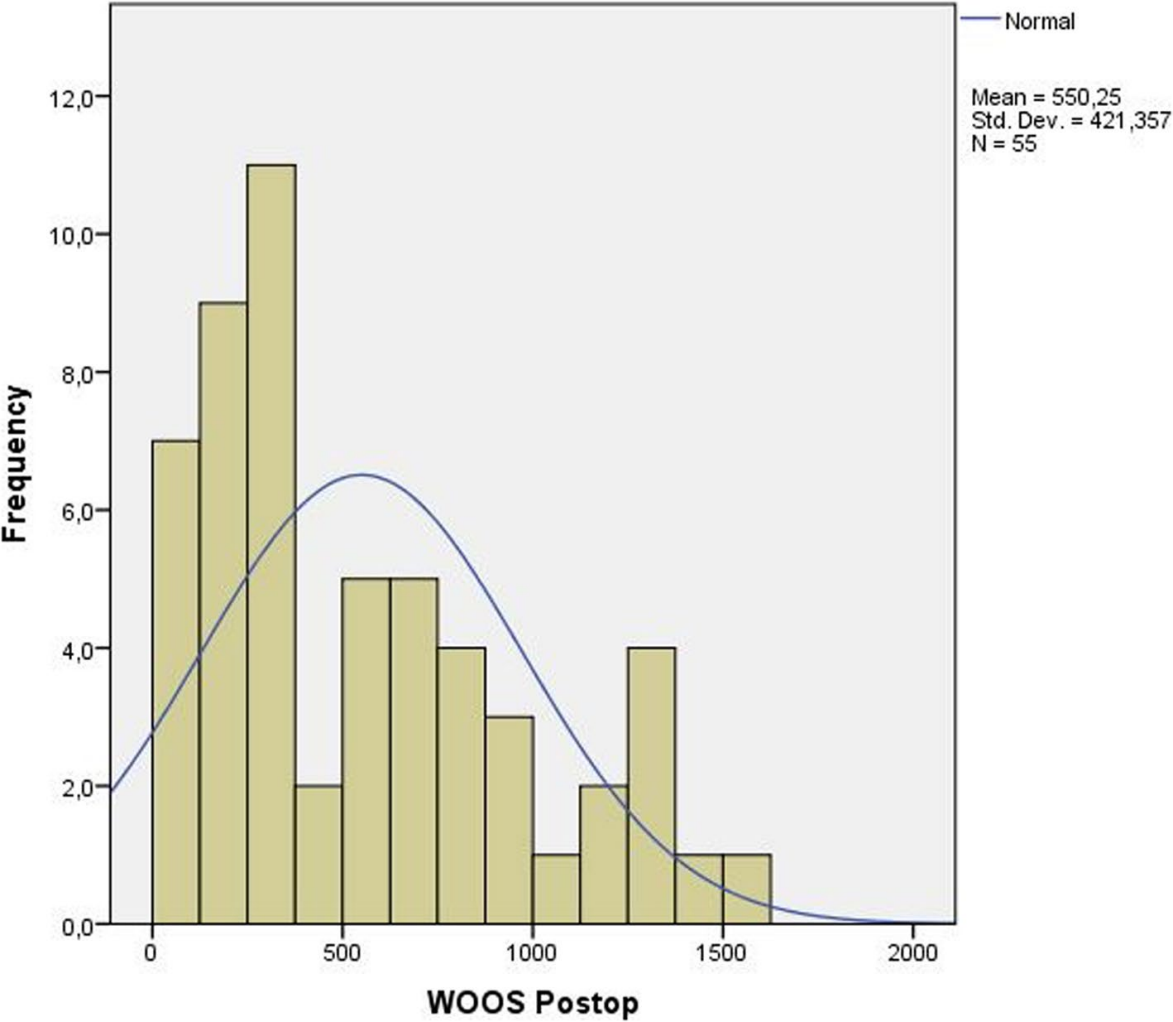
Postoperative WOOS scores presented as a histogram. X-axis=WOOS-score, Y-axis= Number of subjects

Histograms for preoperative and postoperative WOOS scores were plotted (Figs. 1 and 2) to show the normal distribution of the scores. The postoperative group had a higher standard deviation (SD = 421.4) compared to the preoperative group (SD = 239.0).

### Minimal detectable change, and minimum clinically important difference

The MDC for WOOS% was calculated to be 10.2% from the registry population. The MCID WOOS% was defined as 8.2% in the registry population.

### The PROM performance over time in the SSAR

**Fig. 3:**
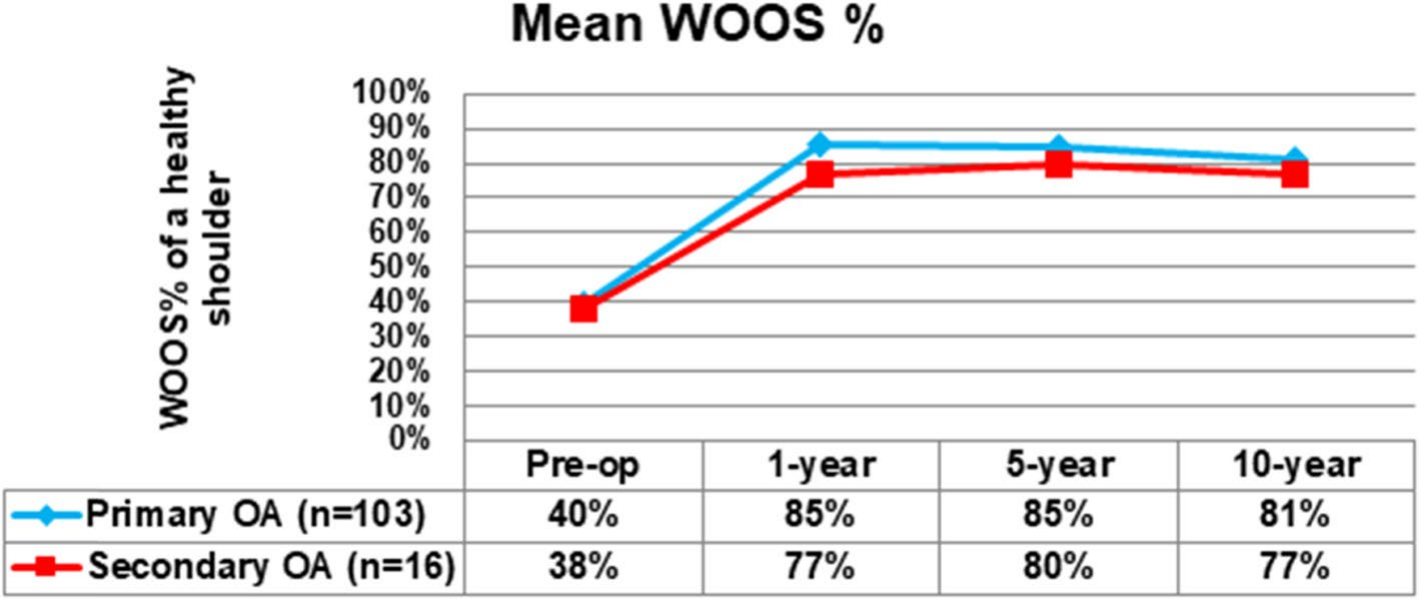
Development of mean WOOS% over 10 years. OA=osteoarthritis

**Fig. 4:**
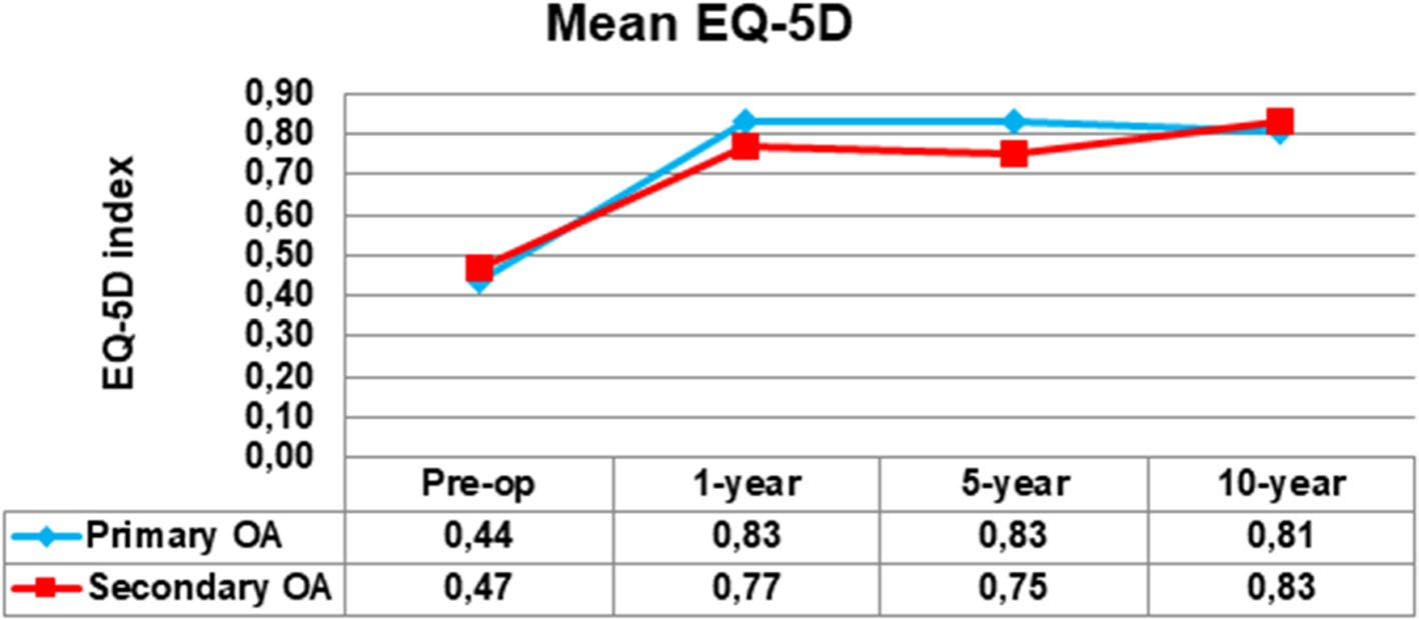
Development of mean EQ-5D over 10 years. OA=osteoarthritis

For the 119 shoulders with all four PROM assessments available, the development over time was stable and improvement from preoperative levels were substantial for both primary and secondary OA (Figs. 3 and 4). Seven implants had been revised: four between 1 and 5 years, and 3 shoulders later than 5 years after the primary procedure.

## Discussion

This study was made to test the validity of the Swedish translation of WOOS. In our study, convergent criterion validity was tested by correlating WOOS to CMS, OSS and EQ-5D. The correlation between WOOS and CMS was high, which is in accordance with the original version in English [1], as well as with the results from a study of the validity of the Danish version of WOOS [18]. The correlation between WOOS and OSS was also high, and our results show that the Swedish version of WOOS is valid compared to both these widely used shoulder-specific scoring systems.

The material in this study was collected from three earlier studies from our hospital. It should be noted that the patients have undergone different types of shoulder arthroplasty, either total shoulder arthroplasty or humeral resurfacing. The two procedures are similar with regard to surgical approach (usually by deltopectoral incision) and postoperative protocol. Total shoulder arthroplasty, however, must entail a larger surgical trauma to the shoulder joint, as both the humeral head and glenoid are prepared and exchanged with implants. Postoperative progression and convalescence show small differences between the procedures [19], but we think it is unlikely that these differences would affect the comparisons and validation of PROMs. In addition, our study does assess the performance of the scores, not the differences between different implants.

The correlation between WOOS and EQ-5D 3L was higher than we expected. In validation studies for the original English version and the Danish version, correlations were made against the general health measure SF36 with 36 items, instead of the EQ-5D. In both studies, the correlations between WOOS and SF36 were shown to be poor [1, 18].

In addition, we examined correlations between each separate domain of WOOS and EQ-5D in this study. The best correlation was seen between the physical domain of WOOS and EQ-5D. This might be explained by the emphasis on pain in the physical domain of WOOS, and that pain also reflects in the EQ-5D to a large extent. The weakest correlation was seen between the emotional domain of WOOS and EQ-5D.

Comparing WOOS domains and the clinically examined items of CMS, we found that the correlations for the total score were 0.53. The highest correlation was seen for the physical domain of WOOS, which may be expected as the physical domain of WOOS covers the same type of issues that a physical examination does. As noted earlier, one difference between WOOS and CMS is that WOOS only contains patient-reported questions, whereas CMS includes questions that necessitates a clinical exam.

Content validity was analyzed with the floor and ceiling effects. There was no floor and a small ceiling effect for total WOOS. There were adequate floor and ceiling effects in some of the domains, the highest being postoperative ceiling effects in the emotions domain. This is in accordance with the results from other articles on validation of the Western Ontario shoulder instruments [7, 18, 20].

Lack of preoperative floor effect is a good property of WOOS, and makes the score sensitive for not only bettered, but also worsened, symptoms. The small postoperative ceiling effect we consider to be acceptable, indicating that some patients reported that they were free of all symptoms after the surgical treatment. This means that they will not be able to report any further improvement in a later assessment. This may be considered as a weakness, and that other measures are needed to assess shoulders that are free of symptoms, even if it still is possible to detect worsening of symptoms with future WOOS measurements.

Analyzing reliability, CA was shown to be excellent in all the domains and for total WOOS in the postoperative group as well as when combined with the preoperative group. CA for total WOOS was very high (0.95), which could indicate that some items are redundant. However, as CA normally increases with the number of items in an instrument (WOOS has 19 items), this might be a contributing factor for the high CA value. Therefore one should be cautious since it may be questionable to compare CA scores between scoring systems whose number of items differs [21]. The number of items may be of importance in item reduction and construction of a new scoring system, but this is not a variable that is possible to influence when analyzing an already established score.

In the preoperative group lower CA values were seen with separately analyzed domains, and the sport and lifestyle domains both had CA values below 0.7. The emotions domain had a CA value of 0.72 and thus graded as adequate. The Physical and Emotional domains both had CA values above 0.7.

Responsiveness to change was analyzed by calculating ES as well as SRM. All four scoring systems showed high ES, all exceeding 0.8. The WOOS score had the highest ES at 2.52, and an SRM of 1.43. These results were similar to the results shown in the study on the Danish translation of WOOS [18], which reported an ES of 2.32 and an SRM of 1.41 for the WOOS score. Support of a high responsiveness for WOOS in shoulder arthroplasty, as well as an excellent correlation with the American Shoulder and Elbow Surgeons score, is also shown in another recent study [22]. In the original WOOS article [1], as well as in the previous Swedish validation of WOOS in patients with subacromial pain [7], only the SRM for the different scores was presented. In these studies, the SRM were 1.20 and 1.91 respectively. We believe that the results give support to the notion that WOOS is a responsive instrument in a clinical setting.

When plotted as a histogram (Figs. 1 and 2), the preoperative scores come closer to a normally distributed curve than the postoperative scores. This could be explained by the large number of good results in the postoperative group and is also reflected by the occurrence of a small ceiling effect. The difference between the ES and SRM for WOOS in our study is an effect of the much larger standard deviation seen in the postoperative group compared to the preoperative; the reason being that ES is calculated using the SD from the preoperative scores, and SRM is calculated with SD from the postoperative change in scores.

The MDC and MCID was found to be at the level of previous estimates for WOOS, with a 10% change or difference as the minimum of clinical relevance recommended for WOOS% [23].

We find it notable that EQ-5D 3L, a general health measure, performs so well compared to shoulder-specific health measures. EQ-5D 3L was shown to be highly responsive for change (ES = 0.82, SRM = 0.86) in patients with glenohumeral OA. EQ-5D 3L provides no possibility to study specific shoulder-related problems and cannot replace WOOS as a shoulder evaluation tool. However, our results suggest that EQ-5D 3L adequately reflects disease-specific QoL in patients with glenohumeral OA. The time and effort needed to complete the EQ-5D 3L questionnaire is less in comparison to the WOOS questionnaire.

The outcome of the treatment, as measured by the PROM used in the SSAR are considered stable over time. There is a slight decrease in the overall results at 10 years, but lower than MDC and it may be difficult to determine if a change is related to the implant performance or a result of increasing patient age. The possible need for an age adjusted WOOS will have to be studied separately. The lack of a clinical examination in WOOS might be regarded as a weakness of the score. However, evidence that WOOS adequately covers these questions could improve evaluation of patients with glenohumeral OA, and save resources, and should be further studied.

One strength of this study is the correlations of WOOS made to both CMS and OSS. CMS is a well-established and widely used shoulder score, and we think it is an important correlation to be made in the validation process of any shoulder score. The correlation to OSS is important since OSS is used in other shoulder arthroplasty registries. This can be of value when comparing results from different registries. The patient cohort was limited but could be considered as useful for the planning of future studies of comparisons of PROM outcome. We also could demonstrate the real performance of the PROM over time, in use for a 10-year follow-up within SSAR. No test–retest analysis was performed within this study, which we consider to be a weakness. A test–retest analysis of the Swedish translation of WOOS might be a subject for a future study to validate the score within the registry.

## Conclusion

The Swedish translation of WOOS is valid, reliable, and responsive for use in a clinical setting for patients with glenohumeral osteoarthritis treated with shoulder arthroplasty, and we regard it as an appropriate instrument for use in the Swedish Shoulder Arthroplasty Registry.

## Data Availability

The datasets used and/or analyzed during the current study are available from the corresponding author on reasonable request.

## Abbreviations

CA: Cronbach’s alpha
CMS: Constant Murley Score
ES: Effect size
EQ-5D-3L: Euro quality-of-life 5 dimensions 3 levels
MCID: Minimum clinically important difference
MDC: Minimal detectable change
OA: Osteoarthritis
OSS: Oxford Shoulder Score
PCC: Pearson’s correlation coefficient
PROM: Patient related outcome measure
QoL: Quality-of-life
SCC: Spearman’s correlation coefficient
SEM: Standard error of measurement
SL: Satisfaction level
SRM: Standardized response mean
SSAR: Swedish shoulder arthroplasty registry
WOOS: Western Ontario Osteoarthritis of the Shoulder score
